# Supplementary material for: Methods to predict the timing and status of biological maturation in male adolescent soccer players: A narrative systematic review
Source: PLoS One. 2023 Sep 8;18(9):e0286768. doi: 10.1371/journal.pone.0286768 (PMC10490883; doi:10.1371/journal.pone.0286768)
Supplement: Attachment — Submitted filename: Methods to predict the timing and status of biological maturation in male adolescent soccer players- A narrative systematic review_Peer Review .docx [file pone.0286768.s001.docx]

Methods to predict the timing and status of biological maturation in male adolescent soccer players: A narrative systematic review.

Overall, this is a very well drafted manuscript with a high level of attention to detail, and the authors should be very proud of their efforts. The methodology is well constructed and logical, and it is clear that a lot of time and effort has gone into this manuscript. Youth football and in particular growth and maturation needs more of this type of high-quality research, since it’s a very important topic. I would be happy to accept the paper for publication if consideration is given to some of the below suggestions.

Not sure if I have missed something, but are some of the tables not referred to in the text of the manuscript? Maybe check this and make sure every table is mentioned.

Introduction

In general, this section is a little long, and could be more targeted to the aims of the paper. From my personal perspective at one stage I thought the paper was going to go in the direction of training load and maturity. While training load is a factor in youth injury, you are evaluating methods to predict timing and status of biological maturation. With this in mind, maybe you could consider some re-working of this section?

Line 57 maybe mention psychological in relation to soccer specific skills

Line 107/108 could you give some details here on what are the negative implications. Many are listed in the systematic review by Jones et al, which you have referenced.

Materials and methods

The search is almost a year old I would definitely suggest re-searching again as there might some new research that could be used.

Inclusion/exclusion criteria (PICOSS)

Line 266/229 would a table not be a better choice vs text?

Line 230 you refer to the EPPP here, but not all of your papers are from EPL academies. I would assume that not all of the readers of this paper would be from clubs working under the EPPP framework – I would remove this.

Line 233/237 would a table not be a better choice vs text?

Check table 1 and make sure that the wording is the same as Line 227, if you decide text is best for this paper.

Data analysis

Is it possible to calculate I^2^ to provide a number for the heterogeneity? The reference for this calculation is in the review you have referenced by Jones et al.

Narrative synthesis of findings

May be consider using a table to replace some of the text here.

Table 3

What is the reason for some of the responses being in bold?

Discussion

I have seen brackets next to brackets, please check this section and correct this, for example Mirwlad (2002, [mean range = 0.6 years]).

Table 4

The data heading could use more detail here, this would make it easier for the reader.

Limitations

Line 597/598 could you provide more details as to why longitudinal studies are a better choice?

Line 624/625 were any studies potentially suitable, but were excluded due to language, if not mention it or was the search function only for English language?

Applied implications

Line 681 does your group have any suggestions of how this can be achieved.

Suggestions for future studies

Could more direct information be provided in this section, it very generic. Providing some specific suggestions will definitely help this paper to be referenced in the future, which it deserves.
