# Supplementary material for: Methods to predict the timing and status of biological maturation in male adolescent soccer players: A narrative systematic review
Source: PLoS One. 2023 Sep 8;18(9):e0286768. doi: 10.1371/journal.pone.0286768 (PMC10490883; doi:10.1371/journal.pone.0286768)
Supplement: Attachment — Submitted filename: Response to reviewer.docx [file pone.0286768.s002.docx]

Dear Editor,

Thank you for completing a review of the manuscript entitled: *Methods to predict the timing and status of biological maturation in male, adolescent soccer players: A narrative systematic review* (Manuscript ID: PONE-D-23-06938).

My co-authors and I appreciate your time and feedback in reviewing the manuscript and are pleased you appreciate the value of the research. In accordance with some of your recommendations we have made the following changes to the manuscript and provide a detailed description below of the changes we have made. Following these revisions to the manuscript we consider it now ready for publication.

Once again, thank you for your time and feedback. We look forward to hearing your decision.

Yours sincerely

The authorial team.

| **Reviewer feedback** | **Authorial team response** | **Revisions made to the manuscript** |
| --- | --- | --- |
| **1. “**Not sure if I have missed something, but are some of the tables not referred to in the text of the manuscript? Maybe check this and make sure every table is mentioned”. | The authors appreciate this comment and have referenced all tables within the text. | Tables 1-9 have now been referenced in the manuscript text. |
| **2. “**In general, this section is a little long, and could be more targeted to the aims of the paper. From my personal perspective at one stage I thought the paper was going to go in the direction of training load and maturity. While training load is a factor in youth injury, you are evaluating methods to predict timing and status of biological maturation. With this in mind, maybe you could consider some re-working of this section?” | The authors acknowledge the introduction may be a slightly long. It was the intention to be as broad as possible with regards to the importance and implications of gathering player’s maturational/growth data for areas such as injury risk, bio-banding and training load management. We have amended the introduction to remove references to the EPPP, given that some countries may not be familiar with this concept. Some references have also been removed that refer to the EPP and the references list has been adjusted accordingly. We have tried to focus our implications and background information on injury risk and have reduced the content around training load management. | See introduction section. |
| “Line 57 maybe mention psychological in relation to soccer specific skills” | The authors acknowledge this comment given the context of the sentence. | The word ‘psychological’ has now been mentioned in relation to soccer-specific skills. |
| “Line 107/108 could you give some details here on what are the negative implications. Many are listed in the systematic review by Jones et al, which you have referenced.” | The authors appreciate the need to express some negative implications of injuries within the academy system and have included a further sentence to explain the importance of regular training and games for development and career progression as suggested by Jones *et al.,* (2019). We have corrected this to reflect that for some academy players this can lead to de-selection from the system and potentially a loss of athletic identity. | “Previous research has also highlighted the importance youth soccer players remining ‘injury-free’ during their academy years due to the negative implications of possible deselection and loss of athletic identity” |
| “The search is almost a year old I would definitely suggest re-searching again as there might some new research that could be used.” | The authors appreciate the date of the original search terms is outside of the normal search parameters. We have conducted another search as of May 2023 and can confirm no additional studies were captured apart from Towlson *et al.,* (2023) which although relevant it did not meet our inclusion criteria. The paper by Towlson et al (2023) captures perception of maturational methods by key stakeholders and using a qualitative methodology. | N/A. |
| “Line 230 you refer to the EPPP here, but not all of your papers are from EPL academies. I would assume that not all of the readers of this paper would be from clubs working under the EPPP framework – I would remove this.” | This is a valid point, and we agree the readership may not be familiar with the Anglophile nature of the EPPP concept and framework for English league clubs. Therefore, all references and content to the EPPP have been removed throughout the paper. | All content and references to the EPPP have been removed. |
| “Line 233/237 would a table not be a better choice vs text?” | Yes agree. This will tidy up the manuscript and present a cleaner version for the readership. Therefore, an additional table has been included for the study exclusion criteria. | See Table 2 for study exclusion criteria. |
| “Check table 1 and make sure that the wording is the same as Line 227, if you decide text is best for this paper.” | The authors have ensured that the wording included in the text is the same as the wording used in the PICOSS study inclusion table (Table 1). | See Table 1. |
| “Is it possible to calculate I^2^ to provide a number for the heterogeneity? The reference for this calculation is in the review you have referenced by Jones et al.” | The authors acknowledge the potential to calculate this statistic to express heterogeneity. Unfortunately, in our case, we were unable to calculate this statistic in the same way that in the review by Joens *et al.,* (2019). This review calculated heterogeneity based on injury incidence as a common outcome variable. However, our review included multiple outcome variables with insufficient data to calculate the heterogeneity for individual outcome variables. This strengthens the need for a homogenous approach to research within this area, starting with the collection of commonly established outcome variables. | N/A. |
| “May be consider using a table to replace some of the text here.” | The authors appreciate that a table may be more appropriate to replace the text included here. An additional table has been included for a narrative summary of the included studies. | See Table 3. |
| “What is the reason for some of the responses being in bold?” | The responses in bold are considered the key domains within the assessment as suggested by Shea *et al.,* (2017). These domains are the main contributors towards the assessment of quality for the review. | N/A. |
| “I have seen brackets next to brackets, please check this section and correct this, for example Mirwlad (2002, [mean range = 0.6 years]).” | The authors acknowledge this comment and have corrected the appropriate section. | See Discussion section. |
| “The data heading could use more detail here, this would make it easier for the reader.” | The authors appreciate the need to provide more detail for the heading of this table and have included examples of invasive and non-invasive methods. | See Table 6. |
| “Line 597/598 could you provide more details as to why longitudinal studies are a better choice?” | The authors have expanded on this point and have expressed why longitudinal studies are preferable for this area of research. This extended time period may allow for the pre-pubescent player to tracked and monitored over time. | **“**Longitudinal studies also have the capacity to determine the stability and reliability of some of the estimates and measures of maturation that are produced by these invasive and non-invasive methods (49).” |
| “Line 624/625 were any studies potentially suitable, but were excluded due to language, if not mention it or was the search function only for English language?” | The authors would like to state that the search function was filtered to English language only studies and this has been expressed in the methods section and within the limitations of the study. No relevant foreign language studies were captured. | N/A. |
| “Line 681 does your group have any suggestions of how this can be achieved.” | This is a really good point, and we thank you for picking up on this. The honest answer is that it will be very difficult and is perhaps the basis for a wider conversation between ourselves (i.e., university academics) and practitioners in the industry at some point. We didn’t want to include this in the paper for obvious reasons, but it is likely that the PMA and Premier League has a wealth of maturity-related data dating back to the creation of the EPPP in 2011 from a magnitude of clubs. One suggestion would be for the Premier League to employ researchers to filter and stratify the data collected from all clubs in order to establish how maturity related assessments have changed over the years and how players growth/maturity related data has changed using these methods in order to establish the longitudinal stability of these methods. Better collaboration amongst clubs is needed to come up with a universal solution to the barriers associated with regular maturational assessment of players, which needs to be enforced through governing body initiatives and policies. The cynical side of me suspects the introduction of the EPPP and the increased volume in training hours which has led to an increase in growth/maturity injuries and they may be sitting on a ticking time bomb. I’m not sure what the PL are doing with the data or how clean it is – but I know the PL club involved in this paper are sceptical about the data capture process. | N/A. |
| “Could more direct information be provided in this section, it very generic. Providing some specific suggestions will definitely help this paper to be referenced in the future, which it deserves.” | We agree with this comment and we were not intentionally been obtuse. We have been cautious about this section, based on some of the comments in the section above. We do feel as though future work would benefit from either a consensus statement or practitioner consensus on the most appropriate measurement protocol. We do a lot of applied work across the football industry (UK) and there is some excellent practice and some not so good – some of this is down to resource but mostly it is down to training and education. However, some potential areas for future research have been identified and included in the text. The authorship have included the plans for our next follow up study to the review, in the form of a practitioner- informed consensus statement of maturity-related injury risk factors and the intended aims/objectives of regular maturational anthropometric assessments from an injury prevention perspective. Happy to have a conversation about this if we can target an appropriate international panel (i.e., PL, FIFA, UEFA etc..) | See suggestions for future studies section. |
